# Editorial: Digital information for patient education

**DOI:** 10.3389/fpubh.2023.1211285

**Published:** 2023-05-26

**Authors:** Feng Guo, Xiaofei Zhang, Paul Lee

**Affiliations:** ^1^College of Management and Economics, Tianjin University, Tianjin, China; ^2^Business School, Nankai University, Tianjin, China; ^3^Southampton Clinical Trials Unit, University of Southampton, Southampton, United Kingdom

**Keywords:** digital technology, information management, patient education, online health community, healthcare

## 1. Introduction

With the development of digital technologies, numerous technologies such as online platforms, big data, live streaming, and artificial intelligence are leading the transformation of health/medical information and are bringing new models for healthcare information management (1–3). Digital information can help patients to access health knowledge remotely and benefit patient wellbeing (4, 5). For example, online health information offered by digital technologies [e.g., online health communities (OHCs) and mobile health services] create different ways of healthcare delivery, alter the management of patients' health (6, 7), and provide a boost to the quality and quantity of healthcare services (2), which is beneficial to improve patients' perceived treatment effectiveness (8).

However, research gaps exist in the literature about the relationship between digital health information and patient education and how to provide digital information to educate and benefit patients by improving their understanding. Patient understanding is significant for health information management and patient education due to the fact that only once patients understand the information online can they learn and benefit from the health knowledge-sharing processes (9). As we know, health information usually contains some medical vocabulary, and the patients have difficulty understanding them. Fortunately, digital technologies can offer personalized health information to patients in different organizational types and provision styles. In this vein, the use of digital technologies by providing new approaches for health information sharing (10) may better educate patients and enable patients to better understand their health. This Research Topic aims to explore the role of digital health information in patient education.

This Research Topic focuses on digital information for patient education and underlines original contributions on patients' better understanding the online health information and knowledge by conceptualizing and contextualizing the organization of digital information on health. In the next section, the articles in this Research Topic are introduced. Finally, we will summarize the study with a conclusion.

## 2. Articles in this Research Topic

This Research Topic contains nine articles that have successfully negotiated the standard *Frontiers in Public Health* peer-review process. While the nine articles are quite diverse in terms of topics, theoretical perspectives, and methodologies, they are also related to one or more of the themes identified above. We have summarized the articles and they can be classified into four themes: (1) new digital tools or methods for patient education and health, (2) social media and social networks promoting healthy information and behaviors, (3) the effect of online health community (OHC) and mobile health (mHealth) on healthy lifestyle and health conditions, and (4) physician online information sharing and patient education.

Two studies related to new digital tools or methods for patient education and health. Wang et al. conduct clinical prognosis in cervical spondylotic myelopathy by applying an established predictive nomogram which is grounded in the pre-operative Japanese Orthopedic Association score, maximal canal compromise, and maximal spinal cord compression. According to the results of the predictive nomogram, it improves the prognosis of high-risk patients by helping clinicians and patients to identify and educate high-risk patients. According to a pilot study to evaluate the efficiency of Individualized Reduction of FaLLs-Online, Van Denend et al. find that key stakeholders' feedback is vital for a meaningful process evaluation and an online delivery support program implementation is beneficial to improve the satisfaction of participants and trainers. Individualized Reduction of FaLLs-Online benefits individuals using wheelchairs or scooters with multiple sclerosis, and future iterations are encouraged to offer a program with diverse approaches to fall prevention and management.

Two articles focus on social media and social networks promoting healthy information and behaviors. Bai et al. quantitatively evaluate the quality of English YouTube video content which is an important information source for testicular torsion. Based on 66 videos collected from YouTube, the results show that general information (i.e., etiology, symptoms, and treatment) is the most common video content. While medical or education-related authors contribute a large proportion of videos on YouTube, the quality of the videos remains poor. It is worthwhile to emphasize that during the COVID-19 pandemic, misleading and false information tended to produce health risks to the viewers. In addition, great efforts are needed to improve the quality of videos about testicular torsion. Faus et al. apply a systemic review to 18 academic articles to study the different effects of healthy behavior-related campaigns. The results indicate that only a few high-quality studies evaluate the effectiveness of social campaigns that are disseminated on Twitter. Furthermore, their effectiveness and influence on public health-related behaviors are arguable due to a lack of evaluation of these campaigns, evaluation of them by ambiguous and biased indicators, and ignoring systematic follow-ups over time. Moreover, the identified limitations in this systematic review are beneficial to optimize the paradigm and improve the effectiveness of the communication strategies regardless of there being no strong evidence that Twitter is a suitable medium to rouse public health awareness about behavioral health issues.

Two articles consider the effect of online health community (OHC) and mHealth on healthy lifestyles and health conditions. Zhou et al. draw on social network theory and self-efficacy literature to explore the effect of online health community engagement on lifestyle changes. By applying structural equation modeling with 320 valid questionnaires, the results indicate that OHC engagement and health self-efficacy facilitate healthy lifestyle changes. OHC engagement also has a positive effect on informational support and emotional support which are positively related to health self-efficacy. Furthermore, the mediating effects of informational support, emotional support, and health self-efficacy are also identified. Yuting et al. track the effects of a mHealth intervention on blood pressure control in the context of a low-resource rural. Based on 148 individuals from low-resource rural settings in Hubei, China, the results show that the mHealth blood pressure monitoring intervention significantly improves systolic blood pressure and the mHealth intervention effectively controls systolic blood pressure, waist and hip circumference. In addition, the mHealth intervention was positively and significantly related to self-reported hypertension compliance, self-efficacy and quality of life.

Another three studies concentrate on physician online information sharing and patient education. Guo et al. adopt the attention perspective to investigate the effect of physician online information sharing on patient education and consider the moderating effects of offline expertise and online reputation. On the basis of 61,566 physician-month observations from an online health platform in China, the results indicate that physician online information sharing positively impacts potential patient education while there is an inverted U-shape relationship between physician online information sharing and realized patient education. Furthermore, a physician's offline expertise impairs the positive effect of physician online information sharing on potential patient education and flattens the curvilinear relationship between physician online information sharing and realized patient education. Finally, a physician's online reputation reinforces the positive effect of physician online information sharing and potential patient education. The study makes contributions to the literature on attention theory and information sharing for patient education and has important implications for patients, physicians, and platform managers. Ma et al. investigate the effect of physician-free and paid knowledge sharing on patient engagement including patient visits and patient consultations from the perspective of signaling theory. Based on the obtained sample of 168,377 physicians from one of the largest OHCs in China, the results reveal that both physician-free and paid knowledge sharing are beneficial to patient visits and patient consultations. In addition, physicians' registration duration in OHCs positively moderates the relationships between physician knowledge sharing (i.e., free knowledge sharing and paid knowledge sharing) and patient engagement (e.g., patient visits and patient consultations). This study contributes to the literature on signaling theory in the OHCs context by revealing the effect mechanism of physicians' educational knowledge-sharing on patients' engagement and provides important implications for practitioners in OHCs. Zhang et al. explore the impact of practical benefits, psychological rewards, and perceived connectedness with OHCs on physicians' continuous knowledge-sharing behaviors from the perspective of motivation theory and consider the contingent effect of physicians' online seniority status. The empirical results indicate that the relationships between practical benefits, psychological rewards and physicians' continuous knowledge-sharing behaviors are positive while the relationship between perceived connectedness and physicians' continuous knowledge-sharing behaviors is negative. Meanwhile, physicians' online seniority status enhances the positive relationship between practical benefits and physicians' continuous knowledge-sharing behaviors but weakens the positive effect of psychological rewards and physicians' continuous knowledge-sharing behaviors. Moreover, physicians' online seniority status alleviates the negative effect of perceived connectedness and physicians' continuous knowledge-sharing behaviors. This study also makes contributions to understand the motivational mechanisms on physicians' continuous knowledge-sharing behaviors in OHCs and has significant implications for managers and decision-makers in OHCs.

## 3. Conclusion

Research on digital information in healthcare is rare in terms of how to provide digital information to educate and benefit patients. This Research Topic includes nine articles that address the gap in the literature by conceptualizing and contextualizing the organization of digital information on health to facilitate patients' education and a better understanding of online health knowledge. It also offers practical implications for patients, physicians, and healthcare providers to improve patient education. In addition, digital information for patient education is a hot topic in the field of E-health and there also existed some limitations (e.g., OHC engagement influences lifestyle changes through dynamic processes), which offers opportunities for future studies.

## Author contributions

All authors listed have made a substantial, direct, and intellectual contribution to the work and approved it for publication.
